# First Graders’ Stationary Behavior in Norwegian After-School Programs: A Mixed Methods Investigation

**DOI:** 10.3390/ijerph18041938

**Published:** 2021-02-17

**Authors:** Knut Løndal, Siv Lund, Anders Lund Hage Haugen, Kirsti Riiser

**Affiliations:** 1 Faculty of Education and International Studies, Oslo Metropolitan University, 0130 Oslo, Norway; siv.lund@oslomet.no (S.L.); anders-lund.haugen@oslomet.no (A.L.H.H.); 2 Faculty of Health Sciences, Oslo Metropolitan University, 0130 Oslo, Norway; kirsti.riiser@oslomet.no

**Keywords:** stationary behavior, physical activity, after-school programs, first graders, physical activity play, mixed methods approach

## Abstract

After-school programs (ASPs) might influence the activities and behaviors of children. The aim of the reported study was to investigate how stationary behavior unfolds during ASP time in a sample of Norwegian first graders. A total of 42 first graders from 14 ASPs were observed during one entire ASP day. ActiGraph accelerometers were used to measure the intensity of their physical activity (PA). Children were found to be involved in stationary behavior for 54.9% of the studied ASP time—a median of 79.5 min (IQR = 62.0). However, there was considerable variation among the children in the sample. Most stationary behavior—63.5% of all stationary behavior during ASP time—was accumulated when the children were *sitting* indoors. The proportion of stationary behavior was significantly higher indoors than outdoors, during adult-managed time than child-managed time, and during time spent together with other children than time spent alone (*p* < 0.05). In child-managed physical activity play outdoors, stationary behavior commonly occurred during short periods of *standing still*. Stationary behavior was usually rapidly broken up by longer periods of PA. Stationary periods involved activities in close relationship with other children and appeared to be important for social interaction and friendship building. The researchers suggest that ASP staff members should actively promote physical activity play that breaks up sedentary time and replaces some stationary behaviors with PA, especially among the least active children.

## 1. Introduction

In many countries, young children attend after-school programs (ASPs) for a significant portion of their time each day [1,2,3,4]. Hence, there is need for research-based knowledge about how ASPs contribute to children’s lives. During the last few decades, this knowledge field has seen an increase in the number of research studies conducted, with researchers studying how children’s ASP attendance affects their health, well-being, development, and learning [5,6,7,8,9]. Among other aspects, researchers express concerns about the opportunities that children have for PA in ASPs and whether they accumulate too much stationary behavior time while attending them. In this article, we address these concerns and explore how children’s stationary behavior evolves during the time they spend in ASPs.

Stationary behavior can be contrasted to physical activity (PA) [10]. PA is characterized by type of activity, frequency, intensity, and duration, comprising bodily movements that require energy expenditure [11,12]. A great number of studies have documented that regular PA in children benefits their health and well-being [13,14]. Health recommendations state that children should be engaged in PA at moderate or vigorous intensity levels for at least 60 min per day [12,15]. Versatile PA among children is also found to be beneficial from a learning perspective—studies indicate that PA can positively influence academic performance of children and promote social behavior [16,17]. Other studies document that PA that comprises varied activity types at various intensity levels enhances the development of motor competencies and the learning of movements in children [18,19,20]. Children’s movement capability is a primary mechanism underlying PA [21], and the development of motor competencies is necessary for ensuring a physically active lifestyle during youth and adulthood [22]. The term “motor competence” encompasses a person’s ability to perform varied motor actions and to coordinate fine and gross motor skills [23].

Recent research projects indicate that excessive time without PA might play a negative role in children’s lives, especially in combination with screen activities [24,25,26]. According to terminology consensus, such behavior can be called “sedentary behavior” or “stationary behavior” [10]. “Sedentary behavior” is defined as any waking behavior that is characterized by an energy expenditure of ≤1.5 metabolic equivalents (METs) while in a sitting, reclining, or lying posture. Researchers have not reached full agreement about an accelerometer-measured cut-off point defining sedentary behavior; however, a cut-off point of 100 counts per minute (CPM) is often referenced [27,28]. From a health perspective, prolonged periods of sedentary behavior are described as being potentially detrimental, and the recommendation is for them to be limited [29]. “Stationary behavior” is defined as waking behavior performed while lying, reclining, sitting, or standing, without ambulation and irrespective of energy expenditure. The learning of movements depends on a variety of physically active movements that contain a wide range of intensities [18,19,20] and primarily implies non-stationary behavior. Based on evidence that links PA of varied movement types to learning outcomes, researchers have argued that replacing some stationary behavior with PA might improve the development of motor competencies and the learning of movements in children [18,26,30]. Our research project has an overarching focus on motor competencies and learning of movements. Therefore, we address *stationary behavior* rather than sedentary behavior in this article. However, we are aware that various types of stationary behavior might have different learning and health consequences in children. For example, standing might produce considerably more energy expenditure than sitting [10], and learning of fine motor skills might have different potentialities in various behavior types [30]. Hence, our study addresses the unfolding of the different stationary behavior types: lying/reclining, sitting, and standing still.

It is challenging for schools to provide children with sufficient PA opportunities during school hours [31,32]. A large portion of school hours is spent with children engaged in stationary behavior in the classroom, including activities that are central for curricular learning in school subjects and for development of cognitive functions. Consequently, extracurricular activities during ASP time provide opportunities for children to engage in PA within the school environment [5,33]. However, despite the fact that behaviors vary widely, research indicates that a high proportion of ASP time is characterized by sedentary behavior. A recent Australian study finds that the time spent in sedentary behavior during the hours of out of school care ranges from 31% to 79%, while screen time accounts for 0% to 41% of that time [34]. Studies from the United States show that young schoolchildren, on average, spend between 40% and 65% of their ASP time engaged in sedentary behavior and that girls accumulate more sedentary time than boys [35,36,37]. A recent Norwegian study finds that sedentary time makes up 40% of the total ASP time, while 44% of it is spent in light-intensity activities [2]. To understand the reason why there is such a high proportion of sedentary time and low-intensity time, there is a need for more research-based knowledge about how this behavior occurs and evolves among children.

Whether institutions are likely to promote PA or stationary behavior depends on both physical and social environments [38]. Research on the characteristics of playgrounds and how they influence children’s activities indicates that playgrounds should provide varied opportunities for children to play at various intensities and that they should be designed specifically for each group of children that is intended to use them [39,40,41,42,43]. This applies to the cognitive and physical challenges that children face, their opportunities to influence the area, their development and skill levels, and their safety. Studies on Norwegian ASPs have investigated how physical activity play influences, and is influenced by, children’s interaction with other children [44,45]. The results show that such play with friends is important in order for them to experience ASP time as meaningful and that it improves their activity levels; in addition, children’s interaction with staff members plays a significant role in their choice of activities [46]. Staff members who implement an initiating and inspiring approach, as well as a participating and interactional approach, have been proven to enhance the tendency of children to engage in physical activity play.

In a recent project, titled Active Play in ASP, the authors of the present article investigated how play-based PA in ASPs contributes to Norwegian first graders’ motor competencies and learning of movements [30]. On average, the children who participated in this study were physically active for 45.1% of the total ASP time and their activities, at all intensity levels, were considered to enhance their development of motor competencies and their learning of movements. However, on average, the children were primarily involved in stationary behavior—consisting of lying/reclining, sitting, and standing still behavior types—for 54.9% of the total ASP time, and girls were found to take part in stationary behavior for a significantly higher portion of time than boys. The present article, which is also based on the data collected for the Active Play in ASP project, focuses on the unfolding of children’s stationary behavior in particular.

The aim of the article is to explore how stationary behavior, including the lying/reclining, sitting, and standing still behavior types, unfolds in a sample of Norwegian first graders during one ASP day. More specifically, we investigate how children’s stationary time in ASP is characterized by (1) type of stationary behavior, (2) outdoor and indoor time, (3) child-managed and adult-managed time, and (4) time spent alone and with others. We hypothesize that more stationary behavior is accumulated indoors than outdoors, during adult-managed time than during child-managed time, and during time spent alone than during time spent together with others.

## 2. Materials and Methods

This article focuses on aspects that concern children’s stationary behavior during ASP time. The Active Play in ASP project, on the other hand, investigated both PA and stationary behavior—hence, we designed the project according to these aims. Consequently, we found it necessary to choose research methods that can separate stationary behavior from *PA* while simultaneously collecting details about both. To achieve this, we took the discernible behavior types highlighted in the definition of stationary behavior—lying/reclining, sitting, and standing still—and the constituting dimensions of PA (type of activity, frequency, intensity, and duration) as our starting point [11,29]. In addition, to obtain qualitative descriptions of PA types and stationary behavior types during one entire ASP day, we collected information about the quantitative dimensions of intensity, duration, and frequency for both behaviors and activities during the observation day.

### 2.1. Study Design

A mixed methods design combines the use of both qualitative and quantitative methods and is deemed fruitful in studies that investigate complex phenomena [47,48]. We argue that such design is helpful for deepening the understanding of stationary behavior among children. Based on recommendations from an overview of research methods offered by Warren et al. [49], we decided to combine direct observation and PA intensity measurement in our study. Direct observation was considered to be suitable for both producing descriptions of children’s activities and collecting measurements about the duration and frequency of behavior types. Following the recommendations by Westerterp [50], we used accelerometers to obtain reliable measurements of PA intensity.

In line with Fetters, Curry, and Creswell’s [48] recommended practices for mixed methods design, we emphasized the integration of qualitative and quantitative approaches in our study design, methods, interpretation, and reporting. We collected qualitative and quantitative data about all children in the sample and conducted our integration analysis by bringing the datasets together after data collection was completed. At the reporting level, we used a contiguous approach by presenting both qualitative and quantitative findings in different sections.

### 2.2. The Participating Children

**Figure 1:**
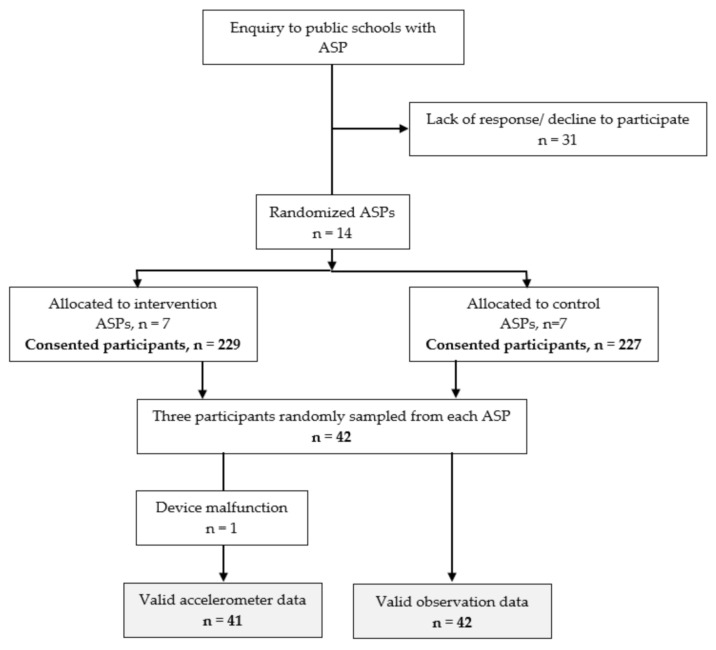
Flowchart for the recruitment of participants.

Information was collected among a sub-group of children who were sampled in the Active Play in ASP intervention study (ClinicalTrials; NCT02954614), as described in the published protocol [51]. A flowchart for the recruitment is shown in Figure 1. ASPs were sampled from three counties in the eastern region of Norway. Leaders of 14 ASPs consented to participate on behalf of their programs. All these ASPs are managed by local municipalities and are located in public school areas. In Norway, parents must pay for their children’s ASP participation [52]. Nevertheless, a very high proportion of Norwegian first graders attend ASPs and it this was also the case in our sample. However, there were differences between the ASPs in our sample. ASPs from both urban and rural areas were represented, and the number of first graders who attended them ranged from 19 at the smallest ASP to 80 at the largest. Although the facilities at the ASPs in our sample differed, all ASPs had access to outdoor areas with varied play equipment. In a recent evaluation report, these variations were found to be typical for Norwegian ASPs [52].

We informed the parents of the first graders attending the selected 14 ASPs about the study, and the parents of a total of 456 children gave consent for their children to participate. Three children from each ASP, a total of 22 boys and 20 girls, were randomly selected for participation in the present study.

### 2.3. Data Collection

As part of the Active Play in ASP intervention study, first graders from 14 participating ASPs wore ActiGraph GT3X accelerometers (ActigraphTM LLC, Pensacola, FL, USA) during their time in ASPs over the course of one week. During this accelerometer-wearing week, a sub-group of 42 children was directly observed for one entire ASP day. Since the accelerometer and observation data should be analyzed together, according to the mixed methods principle of data integration at the interpreting and reporting level [48], only the measures obtained on the observation day were incorporated into the analysis relevant for the present article. Each of the three observers followed 14 children, one at a time, utilizing a pre-prepared scheme designed for PA observation in ASPs [45]. On the first page of the scheme, the observers recorded the beginning and end of each physically active and stationary period, behavior and activity types, activity places, organizational types, and social contexts (see Supplementary Materials). Lying/reclining, sitting, and standing still were considered to be stationary behavior types [29], while locomotory, manipulative, and stabilizing movements were considered to be PA [19]. When a behavior type lasted for 20 s or more, it was checked on the scheme. On the second page of the scheme, the observers wrote qualitative information obtained for each period. The observers were looking for certain predetermined qualitative aspects: what the child was occupied with during each recorded period, how the activity evolved, exact location, who managed the behavior or activity, and whether the child was alone or together with others.

Prior to beginning data collection, the three observers conducted a pilot study in order to reach agreement on the coding criteria to be employed. Additionally, an inter-rater reliability test for the observation scheme was carried out. The results indicated substantial to almost perfect agreement among the observers regarding the coding of PA and stationary behavior, the location in which activities occurred, and how the activities were managed (Fleiss’ kappa (*K*) = 0.62–1.0) [53]. With respect to the social context, the test indicated moderate agreement (*K* = 0.26). The pilot study and the inter-rater reliability test and its results are described in more detail elsewhere [30,51].

### 2.4. Analysis

For the analysis of accelerometer data, we used KineSoft v3.3.80 software (KineSoft, Saskatchewan, Canada). These data were stored in 10 s epochs. We calculated Z-values for skewness and kurtosis for each category of independent, continuous variables—the majority of which did not fall inside the range of ±1.96. Hence, we deemed the distributions of the variables to be skewed [54,55]. As a result, we used medians and interquartile ranges (IQR) when describing continuous variables. The Mann–Whitney U and Wilcoxon signed-rank tests were used when assessing the differences between pairs of continuous data. A significance level of *p* < 0.05 was considered to be statistically significant.

When analyzing qualitative data, we used qualitative content analysis as described by Braun and Clarke [56]. We sought to achieve understanding of the behavior of first graders and placed focus on the contextual meaning of the content. During the coding, we first focused on deductively identifying patterns in the actions of children and considered whether the stationary behavior corresponded with the three discernible behavior types—lying/reclining, sitting, or standing still. Subsequently, we analyzed the qualitative data through an inductive approach and revealed certain typical patterns during indoor versus outdoor time and during adult-managed versus child-managed activities.

### 2.5. Ethical Considerations

Prior to conducting fieldwork, written informed consent was obtained from ASP staff members and guardians of the participating children. The study was approved by the Norwegian Data Protection Official for Research, meeting the requirements of the Personal Data Act (reference number 46008). Prior to each observation sessions, and in line with the recommendations of Backe-Hansen and Frønes [57], we also obtained verbal consent from the observed children themselves. To guarantee anonymity of the participants, fictitious names are used in all publications that make use of the data obtained from the project.

## 3. Results

In this section, we present our findings regarding the participating first graders’ stationary behavior while in ASPs. In the fieldwork, we recorded qualitative and quantitative data during the ASP time of 42 individual children, totaling 6187.2 min. The median age of the first graders in the sample was 6.5 years (range: 1.25 years). On average, they spent 147.3 min in ASPs on the day of observation. Altogether, 71.9% of the total studied ASP time was child-managed and 88.6% of it was spent together with other children. During the fieldwork, we wrote qualitative notes about the 1779 stationary or physically active periods recorded. We intended to obtain accelerometer measures for all participating children during the observation sessions but, unfortunately, one of the accelerometers malfunctioned. Consequently, we obtained accelerometer measures of PA intensity from 21 boys and 20 girls.

### 3.1. Overall Descriptions

The participating first graders were involved in stationary behavior for 54.9% of the studied ASP time and were engaged in PA for 45.1% of that time. The median stationary behavior time was 75.9 min, and girls were found to be significantly more involved in stationary behavior than boys (*p* < 0.05) (see Table 1).

As expected, the median PA intensity level (measured in CPM) during the children’s stationary behavior time was significantly lower than the median of their total intensity level during ASP time. However, the medians for both girls and boys were well above the frequently used cut-off point of 100 CPM [27,28]. The PA intensity level of the total ASP time was lower for girls than boys, although not significantly lower.

### 3.2. Stationary Behavior in Various Settings

In total, we recorded 803 periods of various stationary behaviors. The average duration of stationary periods was 4.2 min, while the duration of the periods varied from 0.3 to 42.0 min. Table 2 shows how the time spent engaged in stationary behavior was distributed between the three characterizing behavior types (lying/reclining, sitting, and standing still), where the stationary minutes were spent, who initiated or managed the activities, and whether the child was alone or together with others.

Most of the observed stationary behavior was accumulated when children were sitting. As much as 69.8% of adult-managed and 70.8% of child-managed stationary behavior in ASPs occurred in the form of sitting. Corresponding percentage values for standing still are 25.5% and 28.0%, respectively. The number of observed standing still periods was approximately equal to those of sitting; however, their median length was significantly shorter (*p* < 0.05). The longest stationary behavior periods that were recorded in the study were those of sitting: 5 children had one period of over 30 min of sitting during ASP time (maximum 70.2 min). Lying/reclining was observed in 9 of the 42 children, and the total time accumulated in such behavior constituted only 1.3% of the overall ASP time. Only two children had lying/reclining periods that lasted longer than 3 min.

As shown in Table 2, significantly more stationary behavior time was accumulated indoors than outdoors, during child-managed time than during adult-managed time, and during time spent together with other children than during time spent alone (*p* < 0.05). However, when taking the proportion of time spent in various contexts into consideration, we found that the proportion of time spent engaged in stationary behavior was significantly higher indoors than outdoors, during adult-managed time than during child-managed time, and during time spent together with other children than during time spent alone (*p* < 0.05). The longest stationary behavior periods also occurred in these contexts. It is worth noting that there were large variations among the observed children in terms of the amount of time spent in these contexts—for example, observed outdoor time ranged from 0.0 min to 190.4 min. Nine of the children spent more than 70% (maximum 96.5%) of their total ASP time involved in stationary behavior, while three children spent less than 30% (minimum 22.0%) involved in such behavior.

### 3.3. The Unfolding of First Graders’ Stationary Behavior

In the field notes, we provided descriptions for all stationary behavior periods recorded. During the subsequent qualitative content analysis, we noticed some typical patterns in indoor versus outdoor time and in child-managed versus adult-managed time, respectively, as well as in the combinations of these two characterizing aspects. Children were rarely alone during ASP time, neither during PA nor during stationary behavior. We recorded only 73 (out of 803) periods during which children were alone when engaging in such behavior—and only 6 of these periods lasted longer than 10 min. The majority of the 73 stationary periods (52) occurred indoors, and the longest of them (with a maximum of 39.8 min) were observed at the end of ASP time, after the closest friends of the observed children had gone home.

#### 3.3.1. Indoors

Most stationary behavior of all stationary behavior during ASP time was accumulated when the children were sitting indoors (63.5%). Indoors, adult-managed time was commonly managed by grouping children and, for the most part, it comprised stationary behavior, with indoor facilities mainly being used for stationary and low intensity activities. A high proportion (75.8%) of adult-managed stationary behavior indoors was accumulated when sitting. Corresponding percentage values for standing still and lying/reclining are 19.0% and 5.2%, respectively. Except for meals, most adult-managed activities were optional for the children. The meal is a daily event at all ASPs studied; however, the way in which it is organized seems to influence the length of the children’s stationary sitting time. At some ASPs, a great emphasis was placed on the meal being an event characterized by discipline during which a staff member would read from a book or talk about what happened at school. In these ASPs, the observed children had sitting periods of more than 20 min during meal time. In one case, the meal lasted for 43.5 min and the observed child spent 39.3 of them sitting. This accounted for a significant portion of the total 64 min she spent in the ASP that day. Some ASPs had freer meal organization, which led to shorter stationary time for the children observed there. At these ASPs, the stationary time during the meal was usually less than 10 min.

The ASP staff also organized music lessons as well as arts and crafts activities. Indoors, much of the stationary time was accumulated during such activities and seemed to be most popular among girls. Child-managed card, board, and certain drama games were also characterized by stationary behavior. Below, we offer an example from a child-managed drama game:

*Marc sits on a sofa, where he is playing with a plastic action figure. His friend soon arrives and sits down next to him. Both are playing with action figures while talking together in low voices. They are very calm and it seems like they are having a close conversation. While this is going on, some other boys are playing actively and noisily around them but Marc and his friend hardly seem to notice them. A third boy joins them. They each have their own action figure and they have a “drama talk” about the figures—they are engaged in a type of war game. They are speaking loudly. Sometimes, the boys get up into a standing position but never for long. They sit on the sofa for long periods and lie on the floor for short periods. Overall, this drama game lasts for 23.8 min.*

The situation described here shows an activity that we often observe among children—they use small play figures and construct fantasy stories that they impulsively live out through the figures. In such drama games and during child-managed card and board games, we often observed rapid shifts between stationary and physically active periods. However, games—such as the one played by Marc and his friend—are frequently seen indoors but rarely last as long.

It is worth noting that we did not observe many behaviors characterized by lying/reclining during ASP time. As mentioned previously, we only observed three prolonged lying/reclining periods. Two of these periods were observed when one child was watching a video film. These periods were the only times that we observed screen time among the children during our ASP fieldwork. Indoors, 78.4% of child-managed stationary behavior occurred in the form of sitting, while 20.2% and 1.4% of indoor stationary time was accumulated in the form of standing still and lying/reclining, respectively.

#### 3.3.2. Outdoors

Outdoors, stationary periods were shorter than indoors and occurred most often during child-managed activities in the sandpit, in short periods during child-managed play, and in short and prolonged periods during adult-managed PA. Additionally, the proportion of sitting was much lower outdoors than indoors—9.4% versus 75.8% of adult-managed time and 44.5% versus 78.4% of child-managed time. The children were usually sitting when playing with digging equipment in the sandpit, most often together with other children. Such behavior demands fine motor skills and often constituted the longest-lasting stationary periods outdoors. Other types of child-managed play outdoors were characterized by social interactions between children and by rapid shifts between standing still and physically active periods, as exemplified in the following situation:

*Linda is together with three other girls in an area with natural elements, such as trees, rocks, stones, bushes, and small cliffs. They are vigorously climbing a “spider net” that hangs between two trees. Suddenly, the girls gather under the net. They stand still and nudge each other as they laugh. They look as if they are sharing a secret together. A while later, they climb the “spider net” again. Sometimes, Linda does the climbing, while, at other times, she stands still and looks at the others. Suddenly, the girls move to another place in the small “forest” area and discover a small puddle of water. Linda stands still for a bit and watches the other girls who are playing with mud. Soon, Linda tries to topple a big stone and the other girls come to help her. Together they manage to topple it and, afterwards, they stand still looking for insects under the stone. They find a beetle. For one minute, they stand still and follow the beetle’s movements with investigative eyes.*

The period described here lasted for 38.2 min. Linda engaged in standing still for about half of that time (19.3 min). However, this total stationary time consisted of 10 separate periods that lasted between 0.5 and 6.8 min each.

Staff members were often close to the children when they were outdoors. However, except for making places and equipment available, they rarely managed the children’s play. When this did sometimes occur, it was interesting to note that the children often accumulated more stationary behavior during such adult-managed activities than during their own child-managed play.

This also applied when the activity was initially based on PA. A typical situation arose during the observation of Samuel:

*John (ASP staff member) announces that a “paintball game” will be organized in the multi-use games area and, together with 11 other children, Samuel decides to join. Together, John and the children place a lot of loose equipment in the games area. Thereafter, John tells the children to gather at one of the goals. Samuel stands inside the goal and waits for John to divide them into teams. The 12 children stand close together and there is a lot of noise. John says that he will not divide them into teams until they have calmed down and everyone is listening. This takes a while. Samuel sits down and waits. He gets up and waits even longer. He seems to get bored, leaves the goal, and finds a ball that he proceeds to play with. A few minutes later, John finally divides the children into teams and the game can begin.*

John’s initiative seems to be popular with the children and a group of girls and boys decide to participate. He is good at including the children in the preparation of the activity but his management style—with an emphasis on structure and discipline—leads to much stationary behavior before the activity actually begins. The stationary periods during the activity itself also turn out to be longer than those usually seen in child-managed play.

## 4. Discussion

Previous research has shown that a considerable portion of the time that young children spend in ASPs is characterized by stationary behavior, and this is often interpreted as a problematic state of children’s everyday lives [5,34]. The results of the present study reveal some issues that might contribute to the nuanced discussion about children’s stationary behavior, especially in terms of where such behavior occurs and in what social and organizational contexts it appears.

A high proportion of the children’s observed ASP time is characterized by stationary behavior. However, such behavior is usually rapidly broken up by periods of PA at various intensity levels, and the total number of stationary periods that exceed 10 min is low. Most of the stationary behavior observed occurs in very short periods during child-managed physical activity play outdoors and is commonly characterized by standing still. Such behavior, with rapid shifts between varied PA types and periods of standing still, is a typical feature of young children’s physical activity play [44,45]. Outdoors, the longest stationary periods—characterized by sitting—are observed during children’s creative play activities with various equipment in the sandpit. These are activities that challenges the children’s fine motor skills with their hands and can thus be considered to be important for learning such skills [20,23]. Overall, outdoor play is shown to enhance a variety of PA types and is considered to be positive for the children’s development of motor competencies and learning of movements [30]. From a health perspective, the recommendation is to limit prolonged periods of stationary behavior [29]. Hence, stationary behavior—characterized by short periods of standing still—accumulated during child-managed play outdoors does not stand out as detrimental. However, the results show very high variations between children in terms of the amount of time spent outdoors, which might be due to the strong emphasis on children having free choice when it comes to activities and activity places in Norwegian ASPs [58]. For some children, potential outdoor time is replaced with indoor time, and the results of the present study indicate that indoor time is characterized by longer periods of stationary behavior in the form of sitting. If we want to reduce long periods of stationary behavior among individual children, especially periods characterized by sitting, it would seem to be relevant to aspire toward increasing the time allotted for outdoor play and facilitating varied activities there.

Indoors, much of the time is characterized by child-managed play. However, this time accumulates more stationary behavior than the time spent outdoors and, unlike outdoor time, sitting is the most common type of child-managed stationary behavior during indoor time. The previously given example of Marc and his friend provides insight into a situation in which boys are deeply engaged in a drama game. Although this game contributes to the accumulation of more than 20 min of stationary behavior, it seems to be valuable to the boys involved. The drama game appears to be a situation in which friendship and unity are developed and maintained. Previous research has shown that the ability to have adapted, social interactions with other children can be developed in institutions such as kindergartens and ASPs [44,59,60,61]. Thus, this situation serves as an example of stationary play that constitutes a positive contribution to ASP time, which should be promoted and facilitated. However, highly valuable interactions and friendship building also take place among peers during physical activity play [44]. Hence, the value of both these play forms in ASP can be argued for. This resonates neatly with the *Norwegian Regulations on Environmental Health Care in Kindergartens and Schools* [62], which highlight that institutions should maintain the varied needs of children for both activity and rest time. It also resonates with the *Convention on the Rights of the Child, Article 31* [63], which emphasizes “the right of the child to rest and leisure, to engage in play and recreational activities appropriate to the age of the child and to participate freely in cultural life and the arts.”

Indoors, staff members play a more managing role in relation to children’s activities than outdoors—they facilitate meals and organize various activity groups. However, our study shows that staff members primarily organize and manage activities that stimulate stationary behavior that implies sitting. Much of this time seems to consist of valuable ASP content. Overall, children are at schools and in ASPs for many hours, so it is considered to be important that they have enough time to eat during their stay. Research also shows that meals are everyday events that are about more than food itself [64,65,66]. A meal represents a situation in which participation, enjoyment of food, and good conversations between children and between children and staff members can be arranged. Consequently, sufficient time to rest and eat is considered to be important, which contributes to the social functions of the meal event.

Music lessons and varied arts and crafts activities organized by the staff also appear to constitute positive ASP content despite their tendency to accumulate stationary behavior during them. These activities, similar to the ones during meals, can provide children with time for close interactions with other children as well as for close conversations with adults. Additionally, such situations might provide opportunities for children to develop aesthetic knowledge, experience creativity [67], and master various fine motor skills [68]. However, it is important to note that much of the observed stationary behavior indoors was accumulated during organized adult-managed activities in the form of sitting and that such activities seemed to be more popular among girls than among boys. When put together with the fact that the girls who participated in the present study partook significantly more in stationary behavior than boys, we find it relevant to ask whether the activities organized by staff contributed to this result. The question also arises whether the offered adult-managed activities appealed most to the least physically active children participating in ASPs. Combined with the established principle of the children’s right to choose activities in Norwegian ASPs [56], this can appear to be problematic—a reinforcement of the stationary tendency in the most stationary children. ASP staff members should reflect on this situation and consider how they can contribute to creating the opposite tendency. An alternative approach might be to inspire the most stationary children to engage in more PA. Based on the results of the present study, however, the most relevant approach would not be to offer adult-managed PA. Sessions with adult-managed PA, both outdoors and indoors, seem to lead to more stationary behavior than those with child-managed play; in addition, the periods of engagement in such behavior are longer. Previous research has shown that an initiating and participatory adult role can be beneficial in promoting physical activity play among children, especially outdoors [46]. This, among other things, involves facilitating play by creating varied play areas and adapting the available equipment as well as by being actively present at the location in which children play.

### Strengths and Limitations

Our investigation was designed to collect both qualitative and quantitative information about the behaviors of first graders during ASP time. This made it possible to record what the children actually do during periods of low and very low PA intensity and to consider whether their behavior during these periods contributes positively or negatively to their overall ASP time. We believe this to be a strength of our study. However, it also has some limitations. Direct observations were conducted by three researchers, separately, which may mean that the behaviors of participating children were recorded differently. As a result, the observation scheme used in the investigation was subjected to an inter-rater agreement test. In addition, the sample size of the study was small. Although typical similarities and differences between Norwegian ASPs are represented in the sample, we must be careful when drawing generalizations from the results of this investigation.

## 5. Conclusions

The results of our investigation, generated through a mixed methods approach, have enabled us to take the discussion beyond a unilateral focus on the intensity of children’s activities in ASPs. A high proportion of the observed children’s ASP time is characterized by stationary behavior. However, the time spent in stationary behavior is usually rapidly broken by periods of PA and the total number of prolonged stationary periods is low. Outdoors, a high proportion of stationary behavior occurs in short periods of standing still during child-managed physical activity play and is a typical feature of such play. From a health perspective, the stationary behavior time accumulated during child-managed play outdoors does not stand out as detrimental. Long-lasting stationary periods commonly consist of activities that demand fine motor skills and can be considered to be positive from a learning perspective. Indoors, stationary periods occur both during adult-managed and child-managed time, most often in the form of sitting. However, the child-managed portion of this time usually involves activities in close relationship with other children and appears to be important for social interaction and friendship building. This study reveals that ASP staff mainly organize and manage activities that stimulate stationary behavior, especially indoors. Adult-managed stationary time also consists of valuable ASP content, such as creative activities, fine motor activities, and close adult–child interactions. However, there is considerable variation among the children in the sample, and some children spend a very high proportion of their ASP time involved in stationary behavior.

### Practical Recommendations

The results of the present study reveal some issues about the behaviors of children in ASPs and, based on the results obtained, we present some practical recommendations. We suggest that ASP staff members actively promote physical activity play outdoors for all children, including the least active children, by taking on an initiating and participatory adult role. This should involve facilitating play by creating varied areas and adapting the available equipment as well as by being actively present at the location in which children play.

## Figures and Tables

**Table 1:** Total time and stationary behavior (SB) time in ASPs, given in median minutes and interquartile range for one observed ASP day, with the intensity given in median CPM and interquartile range. *p*-values are given for the difference between sub-groups.

		Minutes in Observed ASP Time				Intensity (CPM) During ASP Time			
		All (*n* = 42)	Girls (*n* = 20)	Boys (*n* = 22)	*p*-value	All (*n* = 41)	Girls (*n* = 20)	Boys (*n* = 21)	*p*-value
**Total**	Median	146.4	161.5	130.3	0.199	1106	1080	1224	0.167
	Interquartile range	104.5	106.5	106.6		690	627	928	
**SB time**	Median	75.9	93.2	57.2	0.016 *	511	549	510	0.531
	Interquartile range	62.0	41.5	54.0		357	317	320	

* = Statistically significant.

**Table 2:** Stationary behavior (SB) types, place, type of management, and social situation given in total, median stationary minutes for the observed ASP time, *N* = total number of observed children; *n* = number of children observed in the behavior type.

	Total SB Time	Lying/ Reclining	Sitting	Standing Still	Outdoors	Indoors	*p*-Value	Child-managed	Adult-managed	*p*-Value	Alone	Together	*p*-Value
*N*	42	42	42	42	42	42		42	42		42	42	
*n*	42	9	42	40	32	42		40	40		30	39	
Total min. in ASP	6187.2	79.5	2391.8	921.5	2659.2	3528.9		4443.9	1743.2		707.9	5479.2	
Total min. of SB	3392.8	79.5	2391.8	921.5	793.0	2599.8		2305.2	1087.6		270.5	3122.3	
Median min. of SB	75.9	0.0	46.4	20.1	10.7	49.9	0.000 *	41.6	23.2	0.001 *	2.5	69.2	0.000 *
Interquartile range	62.0	0.0	39.5	21.5	25.2	58.8		57.9	23.6		8.2	52.6	

* = Statistically significant.

## Data Availability

The data presented in this study are available on request from the corresponding author. The data are not publicly available due to confidentiality reasons.

## Supplementary Materials

The following are available online at https://www.mdpi.com/1660-4601/18/4/1938/s1, Table S1: Observation scheme: physical activity play in ASPs.
